# Can Better Mother-Daughter Relations Reduce the Chance of a Suicide Attempt among Latinas?

**DOI:** 10.1155/2011/403602

**Published:** 2011-06-28

**Authors:** Luis H. Zayas, Carolina Hausmann-Stabile, Jill Kuhlberg

**Affiliations:** Washington University in St. Louis, Campus Box 1196, One Brookings Drive, St. Louis, MO 63130-4899, USA

## Abstract

National surveys and other research on adolescent Latinas show that adolescent females have higher rates of suicidal ideation, planning, and attempts than other ethnic and racial minority youth. Internalizing behaviors and family conflicts are commonly associated with suicidality in research on adolescents. In the case of Latinas, we explore the connection between adolescent Hispanic cultural involvement, mother-adolescent mutuality, internalizing behaviors, and suicidality. This paper presents data from a study of 232 Latinas, some with a recent history of suicide attempts (*n* = 122). The results show that higher adolescent Hispanic cultural involvement was associated with greater mother-daughter mutuality and thus led to reduction in the likelihood of suicide attempts. The relationship between mother-daughter mutuality and suicide attempts among Latinas is mediated by specific internalizing behaviors (withdrawn depressive). Our findings highlight the positive effect that Latino cultural values have in the relationship between Latina adolescent and their mothers and confirm the importance that internalizing behaviors and the mother-daughter relationship have for suicide attempters.

## 1. Background

 Since 1991, the Youth Risk Behavior Surveillance System (YRBSS) has shown that the rates of suicide ideation, planning, and attempts by adolescent Latinas are higher than those of adolescents of other ethnic and racial groups [1, 2]. Latinas are also more likely to have attempted suicide one or more times in the year prior to the survey (11.1%) compared to Blacks (10.4%) and Whites (6.5%) [1]. In addition, Latina adolescents are known to manifest higher levels of depression and suicidal behaviors than female adolescents from other racial and ethnic groups [3–6]. Not only do Latinas report more persistent feelings of sadness and hopelessness, but they are consistently more prone to ideate, plan, and attempt a suicide. For example, the 2009 YRBSS [1] shows that Latina adolescents were more likely to feel sad or hopeless (39.7%) than any other group of girls (Black, 37.5% or White, 31.1%). Finally, the existing literature has provided ample evidence for the connection between depression and suicide attempts among adolescents [7] and between attempts and completed suicide [8]. 

### 1.1. Hispanic Cultural Involvement, Mother-Daughter Relationships, and Suicidal Behavior

The examination of interpersonal transactions during adolescence between Latina suicide attempters and their families, especially their mothers, can provide important insight into the course of suicidal thinking and behavior [9]. The adolescent years are important in suicidal populations, because during this period the relationship between a mother and her daughter is put to the test [10]. The biggest test is the adolescent's at times confusing desire for autonomy and for connections with her parents. The acculturation differences that US Latino parents and adolescents face influence their interactions and add another layer of complexity to the relational challenges encountered during adolescence [11].

The difference between Latina adolescents and their parents is that youth acculturate faster than parents [12]. These dissimilar acculturation paces may affect the relationship between the girl and her mother [13]. Mothers may wish their daughters to succeed in the mainstream cultural system but at the same time may struggle with the level of autonomy and independence afforded to American adolescents. Daughters may embrace the mainstream cultural customs that are not always endorsed by their mothers. This acculturation dynamic, which often diminishes the mother sense of competency as a mentor to her daughter, may result in the daughter misjudgment of her mother ability to relate, care for, and connect with the girl needs. On another side, mothers may also want their daughters to retain Latino values and cultural practices and may not encourage the daughters movement towards more individual autonomy. Girls who are more involved in Hispanic culture can at least be more attuned to their mothers perspectives than girls who have lower Hispanic cultural involvement [14]. 

Previous findings show that Latina attempters and Nonattempters may be similar in acculturation and familistic attitudes [9]. Attempters, however, have significantly less mutuality and communication with their mothers than Nonattempters [9]. Mutuality refers to the bidirectional exchange of feelings, thoughts, and actions between people in a relationship [15]. Higher levels of mutuality with parents, that is, higher levels of connectedness and communication, are protective factors for suicidal ideation and attempts [9, 16]. The potential pathways from the adolescent Hispanic cultural involvement to her suicide attempts, as mediated by the mother-daughter relationship, require further analysis. 

### 1.2. Family Dynamics, Internalizing Behaviors, and Suicidality

The adolescent interpersonal difficulties with her parents seem to play a central role in depression leading to suicidal behavior [17]. Among Latina adolescents, low levels of family support and high levels of family conflict are associated with more internalizing behaviors [18]. Reducing parent-daughter conflict and fostering closer family ties reduces internalizing behaviors in Latina adolescents and lessens the likelihood of attempting suicide [14]. Achenbach and Rescorla [19] have described internalizing behaviors as encompassing three subgroups to include withdrawn depressive (i.e., prefers being alone; does not enjoy very much; lacks energy; shy or timid), anxious depressive (i.e., frequently crying; feels unloved, worthless; worries), and somatic complaints (i.e., nightmares; constipation; headaches; tired). Our previous examinations of Latina adolescents have shown direct relationships from internalizing behaviors to suicide attempts and from mother-daughter mutuality to suicide attempts [9, 20]. However, these analyses did not disentangle how the three subcategories of internalizing behaviors could mediate the relationship between mother-daughter mutuality and suicide attempts.

In this paper, we explore the relationships between Hispanic cultural involvement, mother-daughter mutuality, three subcategories of internalizing behaviors (i.e., withdrawn depressive, anxious depressive, and somatic complaints), and suicide attempts in adolescent Latinas. We hypothesize that girls reporting higher involvement in the Hispanic culture will have more mutuality with their mothers and that girls with higher mutuality with their mothers will present lower levels of internalizing behaviors and suicide attempts. We also explore which of the three subcategories of internalizing behaviors is the most salient predictor of suicide attempts in our sample.

## 2. Method

### 2.1. Participants and Sampling Design

The data for our analyses come from a cross-sectional study on the sociocultural processes of Latina adolescent suicide attempts. We recruited 122 adolescent Latinas living in New York City who had reported making a suicide attempt in the past 6 months before an interview with our research team. As comparison group, we recruited 110 adolescent Latinas with similar demographic characteristics but with no history of suicide attempts. Suicide attempters were recruited from local hospitals and social service agencies, and comparison group participants were recruited from health and social service agencies in the same communities. 

A suicide attempt was defined as any intentional non-fatal self-injury, no matter how medically lethal, reported by the participant to a friend, teacher, medical staff, or mental health clinician immediately after the attempt was made [21]. Adolescents were excluded from the study if they were outside the study age range (11–19 years old at the time of the interview) or if they were diagnosed with mental retardation or some other serious mental health problem (e.g., schizophrenia). All suicide attempters were cleared for participation by a mental health clinician in order to protect the participants from any psychological harm in the subject matter of the study. Both the assent of the adolescent participants and the consent of one parent were required of all participants. Bilingual and bicultural Master's level clinicians, who were all female, conducted all interviews with the adolescent Latinas. The Human Research Protection Office of Washington University in St. Louis approved all procedures for this study. 

### 2.2. Measures

#### 2.2.1. Demographic and Cultural Covariates

 In this model, demographic data included the girl age at the time of the interview, and the parent educational level (used as a proxy for socioeconomic status). Cultural covariates included the specific Hispanic cultural group(s), with which the adolescents identified, and the girls level of acculturation, as measured by the Bidimensional Acculturation Scale (BAS) [22], which taps both Hispanic and US cultural involvement in two subscales. Both subscales have scores that range from 1 to 4; values over 2.5 are considered “high” for the measured cultural involvement domain [22]. In this sample, the internal consistency alpha for the Hispanic cultural involvement subscale was  .90 and  .83 for the US cultural involvement subscale.

#### 2.2.2. Mother-Daughter Mutuality

We used the Mutual Psychological Development Questionnaire (MPDQ) [15] to measure how attuned girls felt with their mothers. The 22-item scale includes items that assess the relationship across 6 dimensions: (a) empathy, (b) engagement, (c) authenticity, (d) diversity, (e) empowerment, and (f) zest [15]. The total score is the mean score across the items with a score of 1 indicating low mutuality and a score of 6 indicating high mutuality. The internal consistency alpha for the MPDQ reported by the adolescent girls was  .88.

#### 2.2.3. Psychopathology Variables

The adolescents reported their internalizing behaviors using the Youth Self-Report [23], which consists of three subscales (e.g., withdrawn-depressive behaviors, anxious-depressive behaviors, and somatic complaints). Scores ranged from 0 to 16 for withdrawn-depressive behaviors, 0 to 19 for anxious-depressive behaviors, and 0 to 25 for somatic complaints. Higher scores indicated more of the measured behaviors. Coefficient alphas for withdrawn-depressive behaviors, anxious-depressive behaviors, and somatic complaints were, respectively,  .76,  .87, and  .79 for our entire sample.

#### 2.2.4. Suicide Attempter Status

Participants who reported a suicide attempt in the past 6 months before the interview were identified as suicide attempters for all analyses, and those with no reported lifetime history of suicide attempts were classified as nonattempters.

## 3. Data Analytic Strategy

### 3.1. Path Analysis

 After examining bivariate relationships between attempters and Nonattempters and across all other study variables, we used path analysis to test our hypothesized path between the constructs of our study. Using path analysis, we were able to test both direct (i.e., the relationship between the adolescent Hispanic cultural involvement and mother-daughter mutuality) and indirect relationships (i.e., the indirect relationship between the adolescent Hispanic cultural involvement and the internalizing behaviors subscales, mediated by mother-daughter mutuality). We performed the mediating models in Mplus 5.1 [24], using a weighted least squares estimator which allowed for the calculation of both direct and indirect effects of model variables (e.g., mutuality and internalizing behaviors) on the outcome variable. This estimator also produces coefficients that can be interpreted like beta coefficients with continuous outcome variables and probit coefficients with binary outcomes. Model fit was evaluated according to the guidelines presented by Hu and Bentler [25] for the four indicators of model fit: Comparative Fit Index (CFI), Tucker Lewis Index (TLI), Chi Square, and the Root Mean Square Error of Approximation (RMSEA).

Although our data set had minimal missingness, with more than 90% of the cases having complete data across all the variables used in the analyses, we handled missing data with multiple imputation, as supported by Collins et al. [26]. This strategy is shown to reduce bias better than other methods of dealing with missing data, including mean substitution and casewise deletion and works especially well in models with few missing data, as is our case. The majority of the 10% of the missing data was attributed to the parent education level variable, which was only given by parents who were present at the time of the girl interview. We generated 10 imputed data sets using the ICE command created for STATA by Royston [27], taking into account not only variables included in our model but other variables that could be associated with variables in our model (e.g., public or private insurance) or social desirability. The imputed datasets were then analyzed in Mplus [24], using the TYPE = IMPUTED command. 

## 4. Results

### 4.1. Demographics and Bivariate Analyses

Our sample was comprised of 122 (52.6%) adolescent Latinas, who had reported attempting suicide and 110 (47.4%) who had no history of a suicide attempt. Their ages at the time of the interview ranged from 11 to 19 years old (M = 15.5, SD = 2.0). The range of formal education completed by the parents of the adolescents was from 1 to over 17 years, with the average being 10.6 (SD = 3.7) years. 

Most girls identified with one single Hispanic cultural group: Puerto Rican (*n* = 82, 35.3%), Dominican (*n* = 64, 27.6%), Mexican (*n* = 27, 11.6%), Colombian (*n* = 23, 9.9%), and one each from Cuba, Ecuador, El Salvador, Honduras, Nicaragua, and Venezuela. The remaining 30 (12.93%) girls identified with multiple Hispanic cultures, corresponding with the respective cultures of their parents. On acculturation variables, all girls in the sample scored 2.9 (SD =  .07) out of 5 points possible for Hispanic cultural involvement and slightly higher (M = 3.6, SD =  .03) for US cultural involvement. Details of the sample are provided in Table 1.

### 4.2. Bivariate Analyses

 Suicide attempters and Nonattempters showed no differences across nearly all demographic and acculturation variables, with the exception that significantly more Nonattempters identified as Colombian than did suicide attempters (*X* = 7.5; *P* < .01) (Table 1). This difference may have resulted from a sampling bias. Suicide attempters reported significantly lower mutuality with their mothers than did Nonattempters and also reported higher scores than did their Nonattempter counterparts on each of the three subscales for withdrawn-depressive behaviors, anxious/depressive behaviors, and somatic complaints (Table 1). 

Correlations between the continuous variables and suicide attempter status are shown on Table 2. Attempt status was significantly related to mutuality, withdrawn-depressive behaviors, anxious-depressive behaviors, and somatic complaints. Mutuality showed a correlation with each of the subscales of internalizing behaviors: withdrawn-depressive behaviors, anxious-depressive behaviors, and somatic complaints. Finally, all three subscales of internalizing behaviors showed significant correlations amongst themselves: withdrawn-depressive behaviors with anxious-depressive behaviors and with somatic complains, and anxious-depressive behaviors with somatic complaints.

### 4.3. Mediation Analyses

**Figure 1:**
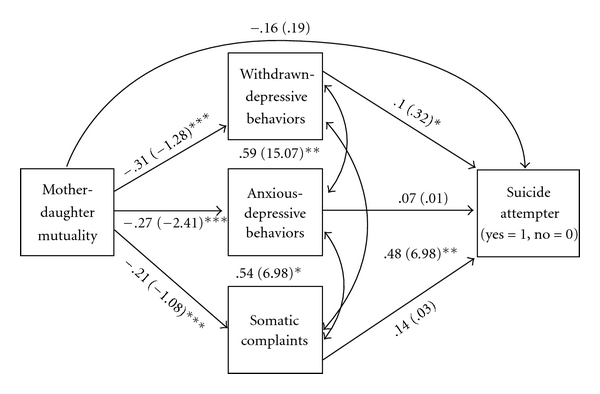
Results of mediation analysis. Note. Standardized coefficients are listed on the model paths, followed by unstandardized coefficients in parentheses. Model fit indices: CFI = 1.0, TLI = 1.0, RMSEA <   .001. Results for covariates were not significant and are not shown. *P < .05, **P < .01, ***P < .001.

The mediation analyses were performed to test the hypothesized path from mutuality through psychopathology variables to suicide attempter status (Figure 1). Demographic and cultural covariates were also included in the mediation model, although their paths are not depicted in the figure for visual clarity. We report the standardized coefficients for the results of the mediation model in the text, which can be interpreted as one standard deviation increase in the outcome variable for each unit increase in the predictor variable; however, both standardized and unstandardized coefficients are presented in the results depicted on Figure 1 (unstandardized coefficients are shown in parentheses).

The final mediation model showed good model fit across the three indices we assessed (CFI = 1.00, TLI = 1.00, RMSEA <  .001). Girls who reported higher Hispanic cultural involvement also reported higher mutuality with their mothers (*b* = .23; *P* < .01). Mother-daughter mutuality had a negative relationship with all three psychopathology variables: withdrawn-depressive behaviors (*b* = −.31, *P* < .001), anxious-depressive behaviors (*b* = −.27, *P* < .01), and somatic complaints (*b* = −.21, *P* < .01). However, the direct relationship between mother-daughter mutuality and attempter status in the mediation model was not significant as it was in the bivariate analyses. Only withdrawn-depressive behaviors were significantly related to suicide attempter status in the path model (*b* = .32, *P* < .05), indicating that girls with higher withdrawn-depressive behaviors were more likely to be suicide attempters, controlling for the other anxious-depressive behaviors and somatic complaints. Neither age nor parent education level of the adolescent Latinas were related to any of the main study variables in the path model. No specific Hispanic cultural group had any significant relationship to any variable in the model, after adjusting for pairwise comparisons.

We tested for significant mediation paths using Sobel's Test (Table 3) to test if mother-daughter mutuality mediated the relationship between Hispanic cultural involvement and any internalizing behaviors subscale and to test if any internalizing behavior subscale mediated the relationship between mutuality and suicide attempter status. Mutuality mediated the effect of Hispanic cultural involvement on withdrawn-depressive behaviors (*Z* = −2.41, *P* < .05), anxious-depressive behaviors (*Z* = −2.17, *P* < .05), and somatic complaints (*Z* = −2.15, *P* < .05). Also, the withdrawn-depressive behaviors mediated the relationship between mutuality and suicide attempter status (*Z* = −2.11, *P* < .05). 

## 5. Discussion

In this study, we set out to analyze a possible mechanism related to adolescents' Hispanic cultural involvement, mother-daughter mutuality, and suicide attempts in Latina teens. Our results demonstrated that girls with higher involvement in Hispanic culture expressed more mutuality with their mothers and in turn lower levels of all three types of internalizing behaviors. We also found that girls who reported having more mutuality with their mothers had less internalizing behaviors, specifically less withdrawn-depressive behaviors, anxious-depressive behaviors, and somatic complaints. We also found that the withdrawn-depressive behaviors were significantly related to suicide attempts, mediating the relationship between mutuality and suicide attempts. Our research builds on past models exploring relational, psychological, and cultural variables involved in the suicide attempts of adolescent Latinas [14, 15] by examining specific pathways from adolescent's Hispanic cultural involvement and mother-daughter relationships to suicide attempts through psychological behaviors.

The girls in our sample that had a higher Hispanic cultural involvement reported higher mutuality with their mothers. Our findings highlight the positive effect that Latino cultural values have in the relationship between Latina adolescents and their mothers. Among Latina teens, when the relationship with their mothers is strained due to their developmental changes [10] and cultural difference [11], Latino culture involvement may play a role in promoting positive family interactions. In our sample, even when culture was not directly related to the girls attempts [9], it had a direct effect on the mutuality between the adolescents and their mothers. Overall, these findings provide additional support for the theoretical and conceptual discussions on the sociocultural and developmental features of suicide attempts among Latinas [28, 29]. 

Other papers have shown that mutuality is directly related to suicide attempts [9] and that unspecified internalizing behaviors are predictors of suicidality [14, 20]. In this study, we find that the relationship between mutuality and suicide attempts is mediated by specific internalizing behaviors (i.e., withdrawn depressive). This offers further clarification on the pathway from family interactions to suicide attempts through mental health profiles.

Although research on adult Latino suicide attempters has found differences among Hispanic subgroups [30], our findings show that no specific Hispanic cultural group had any significant relationship to the variables included in our model. Furthermore, the differences in the rate of suicide attempts among Colombian participants can be explained by sampling issues. 

### 5.1. Limitations and Implications

The generalizability of our findings is limited for a number of reasons. Our sample of adolescent Latinas was not drawn randomly, and all girls live in a larger urban area. Although we present our model as a path model, our data are cross-sectional, and all of the measures used were self-reported. 

Our findings have implications for treatment and prevention of suicide attempts among Latina adolescents. As our results indicate that the suicide attempt incubates within problematic family interactions and relationships and as other family members play a part in influencing the context that set the conditions for the suicide attempt [31], we advocate for family therapy as the first and primary line of treatment. The pressures of negotiating developmental processes and demands of two or more cultures suggest that treatment efforts could also target mother-daughter relationships, perhaps in the context of dyadic therapies. Family and dyadic therapies could enhance parent-adolescent communication by paying attention to reducing conflict, raising mutuality, and communicational quality among family members with different levels of Hispanic cultural involvement. Due to the complexities faced by Latino families negotiating multiple sociocultural systems, any of these interventions should allow for interventions with multiple sessions (>5 sessions). Individual therapy based on interpersonal theory and focused on internalizing behaviors can help Latina suicide attempters to gain problem-solving skills in interpersonal relations. 

The literature has showed that traditional psychotherapy approaches promoting mainstream cultural values (e.g., individualism) may be in conflict with the values of ethnic minorities (e.g., familism) [32]. Our findings point to the importance of including Latino cultural values in the therapeutic approaches offered to Latino families and suicide attempters. The cultural tailoring of mental health treatments is needed to avoid polluting the therapeutic environment with conflictive culturally based beliefs. Moreover, the incorporation of Hispanic cultural values in family and dyadic therapies can help strengthen the members mutuality and fostering psychological wellbeing for the adolescents.

Preventive efforts should target Latino family's level of cultural dissonancy by including parents and adolescents. These interventions should help young Latinas understand their development, acculturation pressures, and their parents cultural expectations. By engaging the parents, the interventions can help them understand their daughters development and cultural perspectives. Ultimately, primary prevention strategies involving Latino parents and adolescents can reduce intrafamiliar levels of conflict and increase the level of mutuality and communication among family members, thus decreasing the likelihood of a suicide attempt. 

## Figures and Tables

**Table 1:** Description of adolescent suicide attempters (*N* = 122) and Nonattempters (*N* = 110).

Variable	All adolescents	Attempters	Non attempter	*P*≤
	M(SD)/ *N* (%)	M(SD)/ *N* (%)	M(SD)/ *N* (%)	
Age	15.5 (2.0)	15.3 (1.8)	15.6 (2.3)	.814
Parent education	10.6 (3.7)	10.2 (3.8)	11.0 (3.5)	.148
Hispanic group				
Puerto Rican	82 (35.3%)	44 (36.1%)	38 (34.6%)	.809
Dominican	64 (27.6%)	38 (31.5%)	26 (23.6%)	.505
Mexican	27 (11.6%)	17 (13.9%)	10 (9.1%)	.178
Colombian	23 (9.9%)	5 (4.0%)	18 (16.4%)	.007
Other	36 (15.5%)	20 (16.4%)	16 (14.5%)	.505
Hispanic cultural involvement	2.9 (0.7)	2.9 (0.7)	2.9 (0.6)	.697
US cultural involvement	3.6 (0.5)	3.5 (0.5)	3.6 (0.4)	.851
Mother-daughter mutuality	4.2 (0.9)	3.9 (0.9)	4.4 (0.8)	.001
Withdrawn depressive	5.9 (3.4)	7.4 (3.1)	4.4 (3.0)	.001
Anxious depressive	8.2 (5.6)	10.7 (5.6)	5.6 (4.2)	.001
Somatic complaints	6.3 (4.0)	7.7 (4.1)	4.9 (3.1)	.001

**Table 2:** Pearson's correlation coefficients between study variables.

	1	2	3	4	5	6	7	8	9
(1) Attempter status	—								
(2) Mutuality	−.28*	—							
(3) Withdrawn depressive	−.45*	−.33*	—						
(4) Anxious depressive	.46*	−.39*	.70*	—					
(5) Somatic	.36*	−.24*	.45*	.61*	—				
(6) Hisp. cultural involvement	−.02	.21	.01	−.01	−.02	—			
(7) US cultural involvement	−.07	−.12	−.12	−.10	.06	−.49*	—		
(8) Age	−.07	−.02	−.04	−.09	−.14	.11	−.13	—	
(9) Parent educucation	−.13	−.02	−.17	−.07	.02	−.19	.20	−.05	—

**Table 3:** Direct and indirect effects in the final path model.

Specific path	*a*	S.E._*a*_	*b*	S.E._*b*_	*Z*
Hisp. cultural involvement → mutuality → withdrawn-depressive behaviors	0.29	0.10	−1.28	0.27	−2.41*

Hisp. cultural involvement → mutuality → anxious-depressive behaviors	0.29	0.10	−2.41	0.71	−2.17*

Hisp. cultural involvement → mutuality → somatic complaints	0.29	0.10	−1.08	0.33	−2.15*

Mutuality → withdrawn-depressive behaviors → suicide attempt	−1.28	0.27	0.09	0.04	−2.11*

Note. *Z*-values are derived using the Sobel Test of indirect effects: *Z* = *ab*/S.E._*ab*_, where *a* = coefficient from initial variable to mediating variable, *b* = coefficient from mediating variable to outcome variable, *ab* = indirect coefficient, S.E. = standard error, and S.E._*ab*_ = √(*b* ^2^ × S.E._*a*^2^_) + (*a* ^2^ × S.E._*b*^2^_).

*P < .05.
